# The outpatient management of hypertension at two Sierra Leonean health centres: A mixed-method investigation of follow-up compliance and patient-reported barriers to care

**DOI:** 10.4102/phcfm.v12i1.2222

**Published:** 2020-06-17

**Authors:** Jenna Herskind, Jon Zelasko, Karlin Bacher, David Holmes

**Affiliations:** 1 Jacobs School of Medicine and Biomedical Sciences, University at Buffalo, Buffalo, New York, United States; 2 Sierra Leone Directory of Partnerships and Development, Jericho Road Community Health Centre, Freetown, Sierra Leone; 3 Department of Family Medicine, Jacobs School of Medicine and Biomedical Sciences, University at Buffalo, New York, United States

**Keywords:** hypertension, medication compliance, Sierra Leone, family medicine, outpatient management

## Abstract

**Background:**

Sub-Saharan Africa faces an increasing burden of non-communicable diseases. In particular, hypertension and its therapeutic control present a challenge and opportunity for health practitioners and health systems within the region.

**Aim:**

This study sought to assess an initiative conducted by two health clinics to begin treatment of hypertension amongst their patient populations by reviewing medication possession rates and documenting patient-reported barriers to care in the provision of chronic hypertension management.

**Setting:**

Two private, outpatient health clinics in Sierra Leone recently beginning hypertension management initiatives.

**Methods:**

A retrospective chart review identified 487 records of patients with diagnosed hypertension and assessed for medication adherence through calculation of medication possession ratios from pharmacy refill data. Surveys were conducted on a convenience sample of 68 patients of the hypertension treatment programme to discern patient-reported barriers of care.

**Results:**

Medication possession rates were found to be less than 40% in 82% (399/487) of patients, between 40% and 79% in 12% (60/487) of patients and 80% or greater in 6% (28/487) of patients. In surveys of individuals being treated by the programme, patients were most likely to cite transportation (81%, 55/68), financial burden (69%, 47/68) and schedule conflicts with work or other prior commitments (25%, 17/68) as barriers to care.

**Conclusions:**

In this newly instituted outpatient hypertensive management initiative, 82% of patients had medication possession ratios under 40%, which is likely to impact the clinical effectiveness of the initiative. The most frequent patient-reported barriers to care in surveys included transportation, financial burden and schedule conflicts.

## Introduction

Sub-Saharan Africa faces an increasing burden of non-communicable diseases that is expected to expand further in coming years because of increasing urbanisation and an ageing population.^1^ Hypertension is a chronic disease, with a global burden of about 1 billion people, leading to 7.1 million deaths annually.^2^ Hypertension is one of the most important risk factors of various types of cardiovascular diseases and is therefore a major modifiable driver of cardiovascular mortality.^3^ A 2015 systematic review and meta-analysis estimated that hypertension prevalence in sub-Saharan Africa as a whole was 30% in all adults (95% confidence interval [CI] of 27% − 34%).^4^ Yet of that population only 18% of patients (CI: 14% − 22%) were receiving treatment for their hypertension and only 7% (CI: 5% − 9%) had blood pressure that was well controlled.^4^ In Sierra Leone specifically, estimated prevalence rates from studies that have conducted population surveys have been reported from 23.4% in Freetown to 14.6% in Port Loko, the second largest town in Sierra Leone, to 27.1% as was reported in Bo, Sierra Leone.^5,6^

Unique barriers to care present themselves to patients and practitioners in countries within the region, and strategies to encourage patient adherence to therapeutic treatments of hypertension are of greater concern. Non-adherence to cardiovascular medication treatment has been shown to be directly correlated with increased risk of mortality.^7^ Patient adherence to specific aspects of hypertension treatment has been assessed through patient surveys at hospitals and health clinics in other countries in sub-Saharan Africa, including Namibia, Ethiopia, Uganda and Nigeria.^8,9,10,11^ These surveys have found levels of adherence ranging from 17% in a survey of 112 hypertensive patients in two city hospitals in Kampala, Uganda, to 61.8% of patients compliant with antihypertensive medications at Jimma University Specialised Hospital in Ethiopia.^9,10^

Through this study we sought to assess and describe the results of the implementation of outpatient hypertensive therapy at two outpatient health clinics in Sierra Leone by (1) assessing the rate of medication adherence through pharmacy refill data and (2) conducting patient surveys to document patient-reported barriers to care in the provision of chronic hypertension management.

## Methods

### Study type

We conducted both a retrospective chart review, to help elucidate patient medication adherence, and a qualitative survey, to better understand patient-reported barriers to care at two private, rural, outpatient clinics in Sierra Leone recently implementing outpatient hypertension management amongst their patient population. The initiative to begin hypertension treatment at each of the healthcare centres began in October 2017 after clinicians and administrative staff at the health centres had noticed that a significant portion of outpatient visits was for somatic complaints relating to uncontrolled hypertension.

Many different methods of assessing the adherence to hypertensive treatment have been described in the literature.^12^ Given the availability of such data at the two outpatient health clinics assessed because of the on-site pharmacy at both locations and the inclusion of refill data into the patient chart, we assessed medication adherence using pharmacy refill data providing an objective and cost-effective measure for estimating clinically relevant adherence.

### Study population

The population included in the study were patients over the age of 18 years and diagnosed with hypertension at two private outpatient clinics in Sierra Leone. The first health clinic, Adama Martha Memorial Community Health Centre, is located in Koidu, the capital of the Kono district, in eastern Sierra Leone and conducts around 20 000 patient visits per year. The second healthcare clinic, Orfonthy Community Health Centre, is situated in a small town in the Port Loko district of north-western Sierra Leone and conducts approximately 7500 outpatient visits each year. Patients at both clinics are from surrounding populations that come to the health centres for primary care or obstetrics services.

### Sampling

Patient records for the chart review were gathered from a hypertension registry compiled by both clinics. All patients diagnosed with clinically significant hypertension between 01 October 2017 and 01 April 2018 and enrolled in the hypertension management programme were included in the chart review. The date 01 April 2018 was chosen as the cut-off date as this allowed the chart review to cover at least 3 months of hypertensive therapy for all patients assessed and to necessitate at least two medication refills and follow-up appointments during the time period of the study. All patients were diagnosed with hypertension during an intake screening for outpatient visits to the clinics. After being diagnosed, all patients were educated about the importance of hypertension as a risk factor of mortality, informed about lifestyle changes that may lower their blood pressure and prescribed anti-hypertensive medication for 1 month. Patients were encouraged to return to the clinic monthly for continued blood pressure monitoring, to assess the tolerability of the medications and to pick up their medication refill for the following month. In total, 530 patient charts were reviewed starting in June 2018. Basic demographic data, such as the date of birth and sex of the patient, the date of first diagnosis of hypertension, the number of follow-up visits attended and pharmacy refill data, were recorded. All patient records with incomplete demographic or follow-up attendance data were excluded from the review, resulting in information from 487 records being included in the final survey. The study design was reviewed and cleared by the University at Buffalo Institutional Review Board on 25 April 2018.

### Inclusion and exclusion criteria

For the retrospective chart review assessing medication adherence through pharmacy refill data, patients were required to be over the age of 18 years, diagnosed with hypertension and diagnosed in between 01 October 2017 and 01 April 2018. Records with incomplete demographic data or incomplete pharmacy refill data were not included in the study, resulting in 43 patient records being eliminated.

All patients who were treated in the programme during a 3-week period in July 2017 and whose charts were included in the retrospective chart review were offered to participate in the qualitative survey. A total of 68 patients agreed to participate in the short survey and the survey was conducted on the clinic grounds.

### Data variables

Data variables gathered during the chart review included patient’s age, gender, date of diagnosis with hypertension, most recent clinic visit, total number of visits to the clinic and medication refill data. Patients selected for the survey were asked two open-ended questions and their responses were recorded and coded afterwards.

### Data collection

Both health clinics included in the study maintain a registry for every patient diagnosed with hypertension visiting the clinic. These registries were initiated in October 2017. Charts are stored separately from patients not diagnosed with hypertension. Every chart on the hypertension registry at both clinics was reviewed. Data collection took place during June 2017 and July 2017 and the results were entered into an Excel spreadsheet that was then rechecked against the charts reviewed.

The perceptions of patients regarding barriers to care were also assessed through a short survey (*n* = 68). Participants were selected from the known population of hypertensive patients as documented by the hypertension registry at the two clinics. Patients on the hypertension registry were recruited during a follow-up appointment or other outpatient service at either of the clinics during the time period that the surveys were administered (June 2018). The survey that elucidated patient perceptions of barriers to care was conducted during the same time period. Surveys were conducted in a separate room in the healthcare clinics by either the study authors or by healthcare providers working at each location. All patients provided consent before the survey was administered. No demographic information was collected for surveys. Patients were asked two open-ended questions that covered their perceptions of barriers to care and the ways in which the clinics could assist them in overcoming those barriers. Responses were recorded and coded into categories by the primary investigator.

### Data analysis

Because of the patient’s procurement of hypertensive drugs at on-site pharmacies during follow-up appointments, we were able to obtain pharmacy refill data, and therefore a quantitative measurement of medication adherence during treatment. Medication adherence was measured using the medication possession ratio, which is calculated by dividing the supply of medication in days by the total number of days from diagnosis to the current chart review. Patients were then categorised into three groups: patients with medication possession ratios above 80% (80% − 100%), patients with medication possession ratios between 40% and 79%, and patients with medication possession ratios below 40%. The cut-off value of ≥80% medication possession was chosen for its use in other hypertensive studies and its validation as a marker of significance in treatment outcomes for a variety of cardiovascular medications.^7,13,14^ Results were compiled in Microsoft Excel version 16.19 and descriptive analytics were performed, including summaries of the basic demographics of patients included in the review, medication possession ratios and the number of follow-up appointments attended by the patients included.

Results from the short surveys were coded by the primary author and then compiled in Microsoft Excel version 16.19. Frequencies of each response for the two questions were calculated and illustrated using basic graphing methods.

### Role of funding source

Data collection was funded by the Glasuaer Externship Fund through the Jacobs School of Medicine and Biomedical Sciences in Buffalo, New York. The study sponsors had no role in the study design, collection, analysis, interpretation of data, writing or decision to submit the article for publication.

### Ethical consideration

This article followed all ethical standards for a research without direct contact with human or animal subjects.

## Results

### Chart review results

**FIGURE 1:**
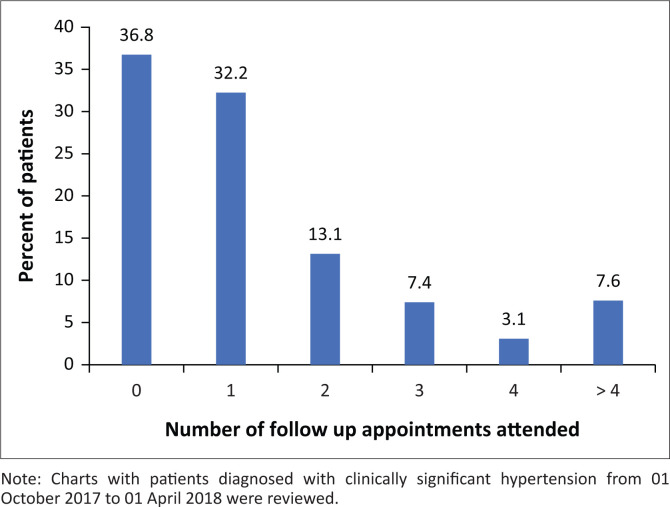
Number of follow-up appointments attended by patients (*n* = 487).

**FIGURE 2:**
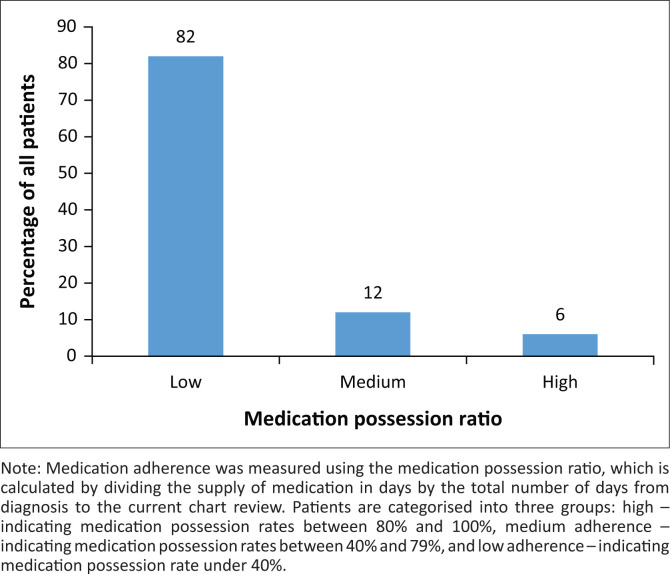
Percentage of all patients with given medication possession ratios (*n* = 487).

Demographics of the patient population captured by the chart review are presented in Table 1. The mean age of the population was 56.9 ± 14.1 years. A majority of records were on female patients (68%, 332/487) and 85% (414/487) were between the ages of 40 and 79 years. Most of the patients (69.0%, 336/487) attended less than two follow-up appointments during the time period reviewed (01 October 2017 − 01 April 2018) as shown in Figure 1). About 36.8% of hypertensive patients (179/487) did not attend follow-up appointments, 32.2% of patients attended one follow-up appointment (157/487) and 30.9% (151/487) of patients attended two or more follow-up appointments (Figure 1). Medication possession ratio was under 40% for 82% (399/487) patients, between 40% and 79% for 12% (60/487) patients and 80% or higher for 6% (28/487) of patients (Figure 2). These percentages did not differ appreciably when broken down by gender, with 82% (127/155) of male patients and 82% (272/332) of female patients having medication possession ratios of under 40%. Similarly, the percentage of patients with medication possession ratios of under 40% remained above 70% for all age ranges in our data analysis.

**TABLE 1:** Demographic characteristics of patients in chart review.

Demographic characteristics	Number	% of participants
**Gender**		
Male	155	32
Female	332	68
**Age (years)**		
≤ 40	54	11
40−59	222	46
60−79	192	39
≥ 80	19	4

### Survey results

**FIGURE 3:**
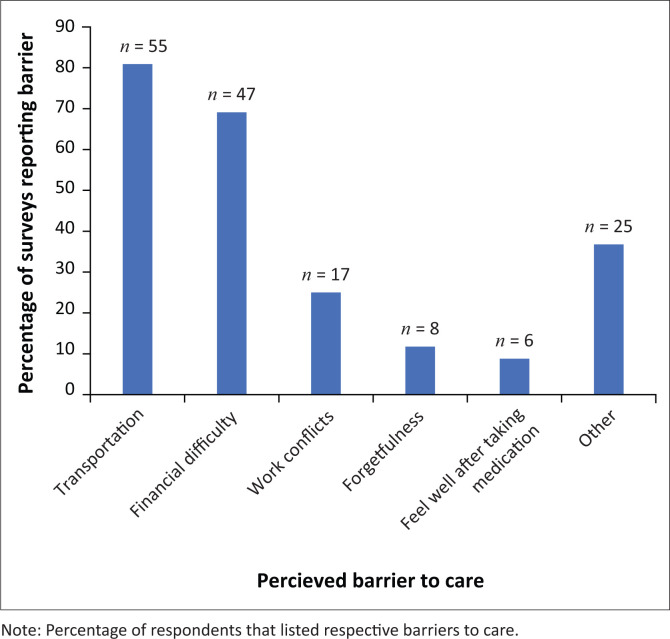
Responses for survey question 1.

The first question the open-ended survey asked was as follows: ‘what challenges do you face in returning to (clinic name) for a follow-up appointment’? Patients were most likely to cite transportation (81%, 55/68) as a barrier to care (Figure 3). Additionally, many patients (69%, 47/68) cited financial difficulty as a problem in returning to the clinics. Schedule conflicts with work or other prior commitments was reported by 25% (17/68) of respondents. Forgetfulness (12%, 8/68) and lack of symptoms (9%, 6/68) were two other challenges that patients reported facing in attending follow-up appointments. A final category of ‘other’ responses was created during coding that included ‘lack of knowledge’, ‘medication runs out too soon’ and ‘rainy season’ as challenges that prevent patients from attending follow-up appointments.

**FIGURE 4:**
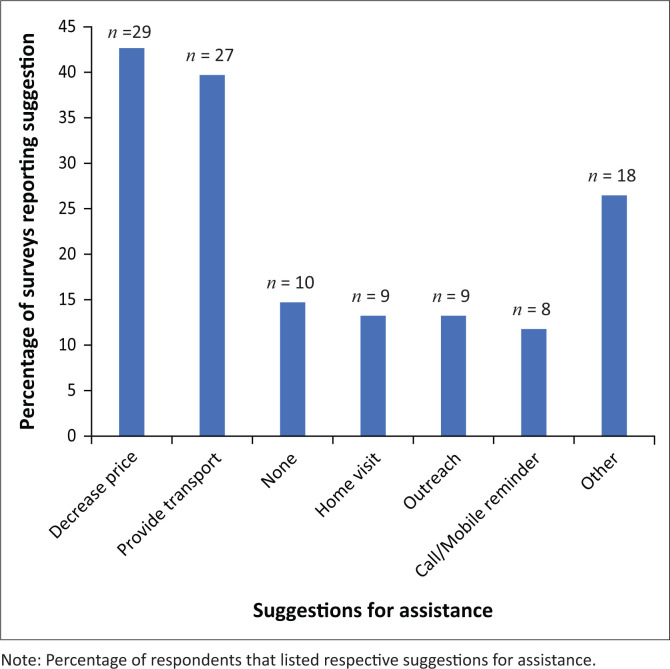
Responses for survey question 2.

The second question in the survey was as follows: ‘what could (clinic name) do to assist you in returning to the clinic for a follow-up appointment?’ The most frequent response, which was offered by 42% (29/68) of respondents, was lowering the follow-up visit price for hypertensive patients, often suggesting that this is warranted as these patients needed to return to the clinic more often than other patients (Figure 4). The second most common suggestion (40%, 27/68) was providing transport to the clinic. Some patients (15%, 10/68) did not offer a suggestion. Home visits (13%, 9/68), outreach (13%, 9/68) and call or mobile reminders (12%, 8/68) were also listed as suggestions for assistance. Other suggestions, such as ‘providing rain gear’, ‘education’ and ‘providing food’, were cited less frequently as ways in which the clinics could assist patients in following up at the clinic.

## Discussion

Our study points to some of the challenges of beginning hypertension treatment in these two clinics as well as hypertension treatment in general. Although it is difficult to compare because of the heterogeneity of assessment tools, populations surveyed and locales, our findings of inadequate medication possession ratios and thereby treatment adherence, with more than 80% of patients assessed not having medication for more than 40% of the time period studied, are congruent with some of the lower estimates of medication adherence previously measured in sub-Saharan Africa that were assessed through the use of the Morisky’s eight-item Medication Adherence Questionnaire.^8,9,10^ Indeed, even a recent review of treatment in high-income countries has shown that less than 50% of patients treated for hypertension have ‘controlled’ hypertension 1 year into treatment.^12^

In the two-question survey that gathered patient-reported barriers to care, issues with transportation and financial burden were reported by a majority of respondents. This is, to our knowledge, the first accounting of patient-reported barriers in Sierra Leone and is in concordance with multiple surveys conducted in different locations in sub-Saharan Africa. Barriers found in other studies, such as lack of funds and equipment shortages at the clinic level, use of traditional medicines and fear of complications, were not reported in our survey.^15,16^

Transportation was the most frequently reported barrier, with 81% (55/68) of respondents indicating it as a barrier to care. The most common method of transportation to the clinic was on paid motorbike. In interviews, patients cited the distance they lived from the clinic, lack of access to motorcycles near their place of residence and the cost of obtaining a motorbike taxi as factors that affected their compliance with treatment. Additionally, when asked what the health centre could do to increase the follow-up appointment compliance (question 2 of the survey), ‘providing transport’ was the second most frequent response (27/68, 40%).

The second most cited barrier to care was financial difficulty (69%, *n* = 47). The cost of a visit to each of the clinics is 40 000 Leones, or approximately $4.75. This cost is further increased by transportation costs to the health centre and the loss of wages incurred with a day spent at the health clinic. In extended interviews with patients, when asked how people felt about the price of a visit to one of the health centres, six of 10 patients said that the price of a health centre visit was ‘very reasonable’. The pricing of a consultation and medications purchased at the clinics compares favourably with other healthcare organisations, both private and public, with much of their revenue coming from private donations rather than patient fees. The flat fee includes consultation with a physician, lab work obtained, intravenous line placement, and medication prescribed. Medication is provided by an American non-governmental organization that prohibits the clinics from charging for medication individually.

However, even with the subsidising of the cost of treatment, this amount represents a likely prohibitive amount on an ongoing basis as, according to recent estimates, 60% of Sierra Leoneans live on less than $1.25 a day, and ‘decreasing price’ (29/68, 42%) was the most widely reported suggestion as to how the health centres could assist in increasing adherence with follow-up hypertension appointments and medication refills.^17^ Some patients surveyed directly identified the nature of chronic treatment, as opposed to more acute, curative services that are often provided, as causing an added financial and logistical burden. Health system features that may be used to pay for preventative care in other locales, or for other diseases such as health insurance or government subsidisation of cost, are largely unavailable amongst the patient population that attend the two clinics for hypertension treatment although evidence suggests that such expenditures may be cost-effective and efficacious.^18,19^ Likely a reduction in the economic cost of hypertension treatment for patients at a larger scale by either decreasing the cost of treatment through task-shifting in care management or by different financing mechanisms may be necessary going forward to accomplish its possible widespread benefit.^18,20,21^

Our survey, reporting only on patient-reported barriers to care, gathered one perspective on what is a complex and multifactorial problem. Although providing insights into one of the most important stakeholders in the issue, there are certainly perspectives that the survey was not able to comment on. For example, many staff at each health centre point to patient education as perhaps the most significant barrier to improve hypertensive treatment adherence. However, patients rarely identified education as a barrier in survey responses. The largely asymptomatic, chronic nature of the problem provides an obstacle to sustained treatment adherence. Other studies in sub-Saharan Africa that have assessed patient understanding of hypertension and its treatment have shown that many patients may discontinue medications because of feeling well, or are uninformed about the benefits of hypertensive treatment with respect to mortality and morbidity over the course of their lives. Additionally, characteristics of clinics and care such as medication availability and clinician behaviour can also influence hypertensive treatment adherence, as shown in a study by Ofili et al. assessing the prevalence, risk factors and barriers to hypertensive care in rural Nigeria.^17^ Team-based interventions that involve the collaborative effort of clinicians, patients and broader health systems that can address barriers in multiple different domains are likely warranted in order to increase the efficacy of hypertensive treatment.

Evidence from other sub-Saharan African countries suggests possible interventions that could be used to mitigate some of the barriers to care reported in our patient surveys. One intervention used a model of Medication Adherence Club (MAC), multiple nurse-led community groups of around 30 patients with non-communicable diseases being managed with daily medications in rural western Kenya.^22^ Outcomes included improved blood pressure control and only a 3.5% loss to follow-up rate at 12 months.^22^ A similar intervention included the implementation of microfinance-linked, community-based groups in rural Kenya that resulted in 70.3% of participants remaining in care through the 12-month evaluation period.^23^

The use of cell phones may prove helpful as well, as suggested by our patients in the second survey question. A study assessing patient perceptions of mobile phone use to increase medication adherence showed positive patient perceptions of mobile interventions amongst stroke survivors in Ghana.^24^ Additionally, one South African intervention used text messaging-based intervention to send reminders to patients to take their medication and return for follow-up appointments.^25^ Qualitative analysis revealed higher levels of patient motivation to make daily health changes in diet and exercise, as well as improved attitudes towards their agency in controlling their diseases.^25^

We are aware of additional limitations in the implementation and broader applicability of our study that have not been previously discussed. Selecting uniquely from the sample of patients who have returned to the clinic for follow-up hypertension appointments for our survey introduces possible selection bias during the short survey given the design of the study. Chart reviews were drawn from outpatients visiting the clinic for ailments other than hypertension and, as such, may represent patients who are healthier than the average hypertensive population and, perhaps, less likely to be compliant with treatment. Additionally, medication adherence is likely overestimated as within the use of a medication possession rate measure for medication adherence is the assumption that patients who have recieved their medication from a dispensary take their medication every day, which is most certainly not the case. Finally, indicators that more directly suggest treatment efficacy, such as the proportion of patients whose blood pressure was under control at the time of the study, were not collected.

Non-communicable diseases, including hypertension and other cardiovascular sequelae, will likely represent a significant proportion of necessary treatment in the years to come in Sierra Leone and similar locales. As such, a shift in healthcare delivery strategies and the financing of healthcare treatments to prevent adverse complications may be needed to effectively deliver new types of care to patient populations. In this article, we present data that indicate barriers to adherence, which likely significantly decrease the efficacy of treatment. Healthcare organisations of similar structures and locales may confront comparable barriers and inefficiencies, and these should be explored and mitigated in order to provide clinically effective care.

## Conclusion

In what is, to our knowledge, the first assessment of hypertension management in the Sierra Leonean context, we conducted a chart review and assessment of patient-reported barriers to care of a newly instituted hypertension treatment programme at two private outpatient health clinics in Sierra Leone. We found a medication possession rate of under 40%, and thereby low patient adherence with treatment, in an overwhelming majority (82%, 399/487) of patients, likely significantly decreasing the effectiveness of therapeutic management of hypertension amongst this population. In the open-ended surveys of patients being treated in the programme, patients were most likely to cite transportation and the financial burden of an already subsidised treatment cost. We propose that a shift in healthcare delivery strategies may be needed when attempting to implement hypertension management programmes in similar localities.
